# *Alhagi sparsifolia* acclimatizes to saline stress by regulating its osmotic, antioxidant, and nitrogen assimilation potential

**DOI:** 10.1186/s12870-022-03832-1

**Published:** 2022-09-21

**Authors:** Abd Ullah, Akash Tariq, Jordi Sardans, Josep Peñuelas, Fanjiang Zeng, Corina Graciano, Muhammad Ahsan Asghar, Ali Raza, You-Cai Xiong, Xutian Chai, Zhihao Zhang

**Affiliations:** 1 Xinjiang Key Laboratory of Desert Plant Roots Ecology and Vegetation Restoration, Xinjiang Institute of Ecology and Geography, Chinese Academy of Sciences, Urumqi, China; 2 State Key Laboratory of Desert and Oasis Ecology, Xinjiang Institute of Ecology and Geography, Chinese Academy of Sciences, Urumqi, China; 3 Cele National Station of Observation and Research for Desert-Grassland Ecosystems, Cele, 848300 China; 4 University of Chinese Academy of Sciences, Beijing, China; 5 CSIC, Global Ecology Unit, CREAF-CSIC-UAB, Bellaterra, 08193 Barcelona, Catalonia Spain; 6 CREAF, 08193 Cerdanyola del Vallès, Catalonia Spain; 7 Instituto de Fisiología Vegetal, Consejo Nacional de Investigaciones Científicas Y Técnicas, Universidad Nacional de La Plata, Buenos Aires, Argentina; 8 Department of Biological Resources, Agricultural Institute, Centre for Agricultural Research, ELKH, Martonvásár, Hungary; 9 Chengdu Institute of Biology, University of Chinese Academy of Sciences, Beijing, China; 10 State Key Laboratory of Grassland Agro-Ecosystems, Institute of Arid Agroecology, School of Life Sciences, Lanzhou University, Lanzhou, 730000 China

**Keywords:** Hyperarid desert, Ions homeostasis, Physiological indices, Saline stress, Xerophytes

## Abstract

**Background:**

*Alhagi sparsifolia (Camelthorn)* is a leguminous shrub species that dominates the Taklimakan desert’s salty, hyperarid, and infertile landscapes in northwest China. Although this plant can colonize and spread in very saline soils, how it adapts to saline stress in the seedling stage remains unclear so a pot-based experiment was carried out to evaluate the effects of four different saline stress levels (0, 50, 150, and 300 mM) on the morphological and physio-biochemical responses in *A. sparsifolia* seedlings.

**Results:**

Our results revealed that N-fixing *A. sparsifolia* has a variety of physio-biochemical anti-saline stress acclimations, including osmotic adjustments, enzymatic mechanisms, and the allocation of metabolic resources. Shoot–root growth and chlorophyll pigments significantly decreased under intermediate and high saline stress. Additionally, increasing levels of saline stress significantly increased Na^+^ but decreased K^+^ concentrations in roots and leaves, resulting in a decreased K^+^/Na^+^ ratio and leaves accumulated more Na + and K + ions than roots, highlighting their ability to increase cellular osmolarity, favouring water fluxes from soil to leaves. Salt-induced higher lipid peroxidation significantly triggered antioxidant enzymes, both for mass-scavenging (catalase) and cytosolic fine-regulation (superoxide dismutase and peroxidase) of H_2_O_2_. Nitrate reductase and glutamine synthetase/glutamate synthase also increased at low and intermediate saline stress levels but decreased under higher stress levels. Soluble proteins and proline rose at all salt levels, whereas soluble sugars increased only at low and medium stress. The results show that when under low-to-intermediate saline stress, seedlings invest more energy in osmotic adjustments but shift their investment towards antioxidant defense mechanisms under high levels of saline stress.

**Conclusions:**

Overall, our results suggest that *A. sparsifolia* seedlings tolerate low, intermediate, and high salt stress by promoting high antioxidant mechanisms, osmolytes accumulations, and the maintenance of mineral N assimilation. However, a gradual decline in growth with increasing salt levels could be attributed to the diversion of energy from growth to maintain salinity homeostasis and anti-stress oxidative mechanisms.

## Background

Of the drylands that cover 41% of the Earth’s surface, 15% are salt-affected [1, 2] and as a consequence, the productivity of many dryland regions, including those with saline soils, are limited by nutrient deficiency [3, 4]. Nitrogen-fixing leguminous trees and shrubs play an essential role in improving dryland soil structure and productivity as they can thrive in nitrogen-depleted soils [5] and have an important bioremediating capacity in areas subject to salinity [6, 7]. Therefore, planting plants capable of adaptation to survival in these conditions (i.e. N-deficient and salinity) is worthwhile but, nonetheless, extremely challenging [8].

It still remains unclear how young leguminous seedlings adjust their metabolism to salinity to ensure growth and survival in the hyperarid saline desert of Taklimakan (NW China). As a consequence of salinity, toxic accumulations of Na^+^ and Cl^−^ occurring in the soil can enter into plants and inhibit metabolism and growth [9]. When salt concentrations increase, ionic imbalance and osmotic stress develop in plants, which can negatively affect their morphology, biomass and biochemical processes [10, 11]. To overcome this, plants have developed a variety of strategies, including (a) the elimination of excess Na + and Cl- ions to the vacuole or older parts to minimize the damage associated with excess salt ions, (b) biosynthesis of osmolytes (soluble sugar, amino acids, and proline) which protect plants salinity-induced osmotic stress and (c) activation of both enzymatic and non-enzymatic and antioxidant defence systems to remove the excess Reactive Oxidative Species (ROS) and protect plants cells from oxidative damage [12–14].

The enzymatic antioxidant mechanisms include changes in the activities of antioxidant enzymes [(superoxide dismutase (SOD), catalase (CAT) and peroxidase (POD)] [15, 16]. For instance, the SOD dismutases O_2_- into H_2_O_2,_ while POD and CAT are responsible for scavenging H_2_O_2._ These antioxidant enzymes have been reported to positively correlate with a plant’s ability to resist salt stress [17]. Plants often undergo a reduction in biomass due to salinity stress, which is linked to decreased carbon assimilation [18, 19]. Plants exposed to these conditions devote more carbon to energy production and to maintaining salinity homeostasis and stress-coping mechanisms than to plant organ development [20, 21]; a good example of this is the increased biosynthesis of stress-relieving biomolecules such as sugar and proline [22–24]. The accumulation and remobilization of photosynthetic products also play a key role in alleviating salt-associated damage in plants and examples include the accumulation of soluble sugar [25], starch [26] and non-structural carbohydrates [27]. Moreover, these products also play critical roles in signalling, osmotic adjustment, ROS scavenging [22] and the regulation of water transport mechanisms [28], as well as photosynthesis through feedback inhibition [22].

Leguminous plants meet nitrogen (N) requirements either by symbiotic N_2_ fixation or by taking up mineral N and they also use a facilitative strategy whereby symbiotic N_2_ fixation can be increased or decreased by reducing the N demand in terms of soil N availability [29]. Soil salinity can affect both symbiotic N_2_-fixation and mineral N assimilation. The extent to which these mechanisms differ between different plant species and environmental conditions remains unclear but research does report that salt ions limit NO_3_^−^ reduction and NH_4_^+^ cell concentration by affecting the activities of NR, GS and GOGAT enzymes [30–32].

The regulation of N metabolism profoundly influences the ability of plants to tolerate salinity stress. However, the relationship between N metabolism and salinity is complex as it depends on the level and duration of salt stress, the plant species, and the quantity, type and form of N available in the soil [33–35]. Interestingly, most studies examining the response of N metabolism to salinity have been conducted on cultivated plants [32, 36], leaving uncertainty as to whether leguminous plants will have similar responses, particularly in drylands [37].

*Alhagi sparsifolia* Shap. is a perennial phreatophyte native to the saline, hyperarid desert regions of northwest China and adjacent countries in Central Asia. It serves various social and ecological services, such as preventing desertification and dunes, reducing salinization, and improving livelihoods. However, anthropogenic activities such as population growth, urbanization, industrialization, overgrazing, over-harvesting, and agricultural expansion threaten its abundance and habitats [38]. Evidence has underlined the urgent need for the revegetation and restoration of *Alhagi* vegetation [39]. Mature *A. sparsifolia* benefits from its deep and extensive root system to exploit and use the groundwater resources. However, extreme environmental conditions persist in the hyperarid desert and can severely affect its growth and metabolism, particularly at the initial growth stages, causing a severe threat to its establishment and survival since its roots have not yet reached groundwater [38]. This scenario makes planting young seedlings for vegetation restoration challenging in a hyperarid and saline desert environment [11]. Therefore, investigating the saline stress adaptation strategies in young *A. sparsifolia* seedlings could provide a theoretical framework for restoring *Alhagi* communities and protecting the fragile ecosystem of the Taklimakan desert. Previously, mature *Alhagi* stands in the natural desert have been studied previously for absorption of toxic salt ions [40] and N acquisition strategies [41], in response to soil factors. Research by Zeng et al. [42] also examined salt ions distribution and photosynthesis in one-year-old A. sparsifolia under different NaCl stress levels but the response of young *A. sparsifolia* seedlings to varying levels of saline stress still remains largely unknown.

The aim of this research was (1) to investigate the morphological and physio-biochemical responses of young *A. sparsifolia* seedlings to different levels of saline stress; and (2) to ascertain its level of saline stress tolerance– based on the interplay between nitrogen assimilation, antioxidant defence mechanisms, and osmolytes accumulation that is used to mitigate the negative impacts associated with an excess of salt ions. Parameters selected for measurement to meet the objectives of the research were shoot and root growth, fresh and dry biomass, chlorophyll pigment concentrations, nitrogen metabolism, osmolytes accumulation, reactive oxygen species production rate, antioxidant enzymatic activities, and root and leaf Na + and K + concentrations.

## Results

### Salt-stress-dependent changes in growth attributes

**Fig. 1:**
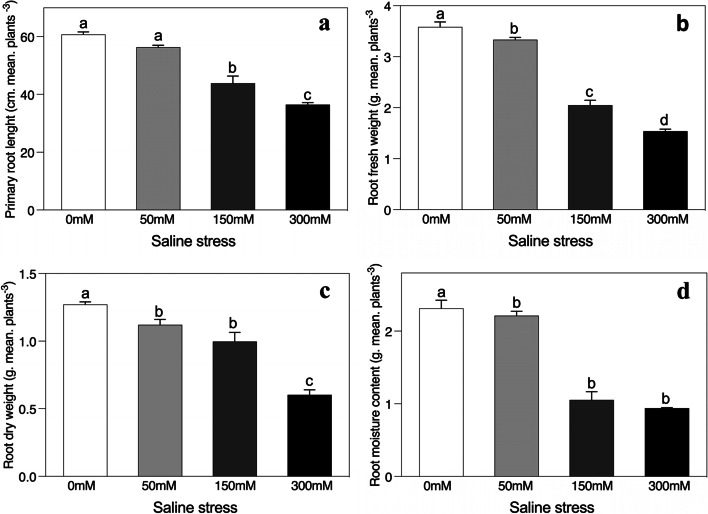
Effects of salinity on (**a**) primary root length, (**b**) root fresh weight, (**c**) root dry weight, and (**d**) root moisture content of *Alhagi sparsifolia* at days 90th of exposure to a different gradient of salinity (0, 50, 150, and 300 mM). Bars represent standard errors of the means (*n* = 3). According to Duncan’s Multiple Range Test, different letters indicate significant differences among the treatments at *P* < 0.05

**Fig. 2:**
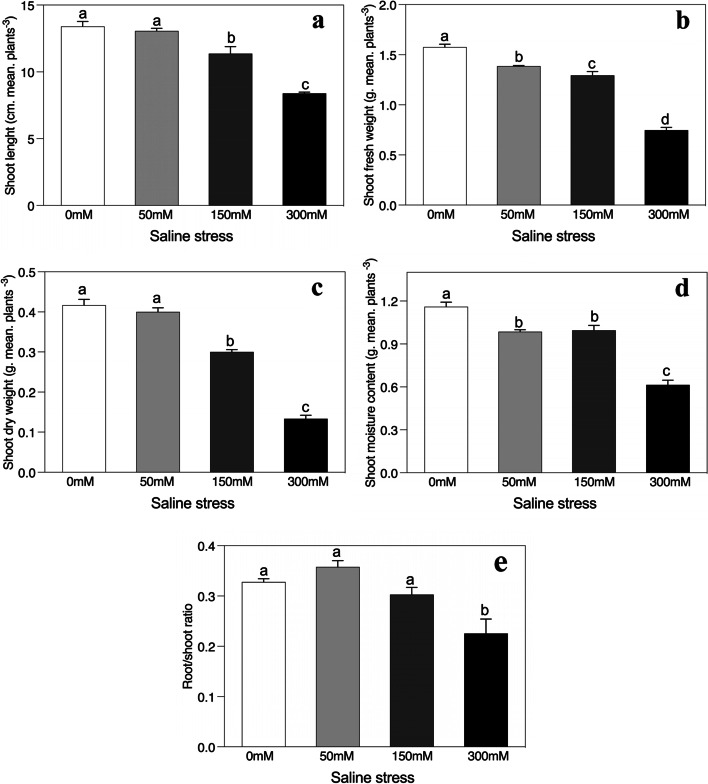
Effects of salinity on (**a**) shoot length, (**b**) shoot fresh weight, (**c**) shoot dry weight, (**d**) shoot moisture content, and (**e**) root/shoot ratio of *Alhagi sparsifolia* at days 90th of exposure to a different gradient of salinity (0, 50, 150, and 300 mM). Bars represent standard errors of the means (*n* = 3). According to Duncan’s Multiple Range Test, different letters indicate significant differences among the treatments at *P* < 0.05

Salinity stress significantly decreased the growth attributes of *A. sparsifolia* plants compared to the controlled conditions (Figs. 1a-d and 2a-e). The fresh root weight, dry weight, length and moisture content were significantly twofold inhibited as the saline stress rose to 300 mM (Fig. 1a-d). Similarly, saline stress also significantly reduced shoot-related attributes, the maximum inhibition occurring in the highest salt-stress treatment (300 mM saline stress) (Fig. 2a-e). The fresh shoot weight, dry weight, length, and shoot water content decreased 2-, 3-, 1- and onefold, relative to the unstressed control, respectively. In addition, the root/shoot ratio increased at low salinity levels, although, by contrast, it fell at high and intermediate levels (but only significantly at high saline stress) (Fig. 2e).

### Influence of saline stress on chlorophyll concentrations

**Fig. 3:**
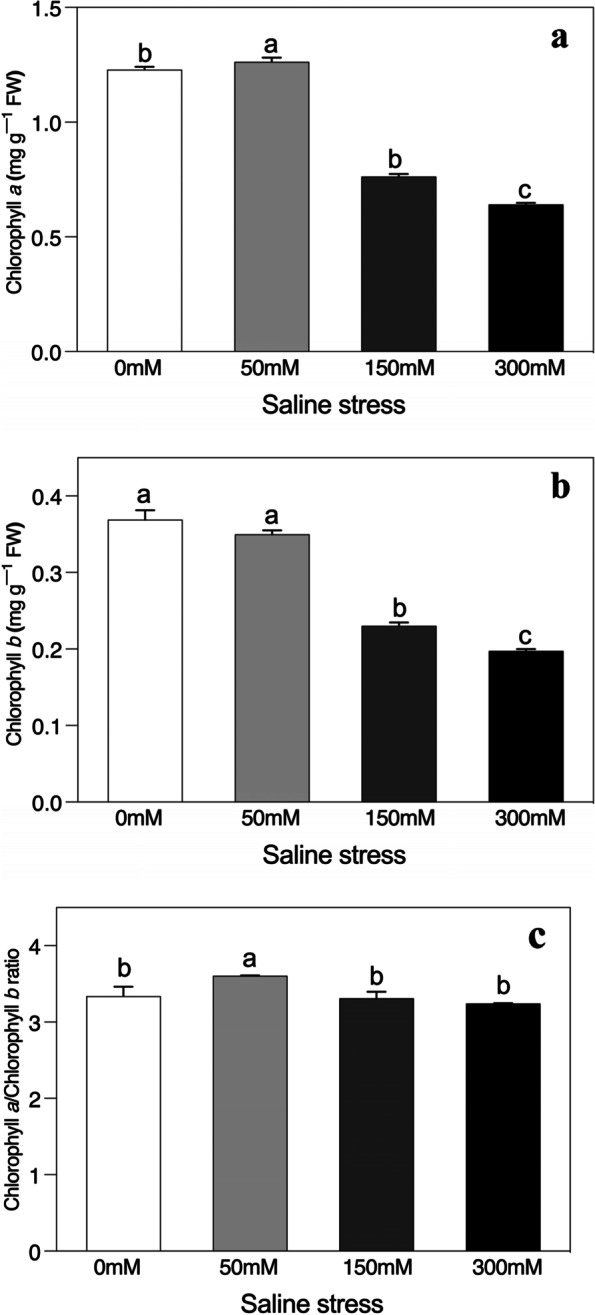
Effects of salinity on (**a**) Chlorophyll *a*, (**b**) Chlorophyll *b*, and (**c**) Chlorophyll *a*/Chlorophyll *b* ratio of *Alhagi sparsifolia* at days 90th of exposure to a different gradient of salinity (0, 50, 150, and 300 mM). Bars represent standard errors of the means (*n* = 3). According to Duncan’s Multiple Range Test, different letters indicate significant differences among the treatments at *P* < 0.05

We observed a slight increase in the concentrations of chlorophyll a and chlorophyll b and in the chlorophyll a/b ratio at low saline stress compared to the control treatment (Fig. 3a-c). Intermediate and high saline stress significantly decreased both chlorophyll a and chlorophyll b concentrations, although the chlorophyll a/b ratio did not change significantly, relative to the unstressed control.

### Effect of salt stress on MDA and H_2_O_2_

**Fig. 4:**
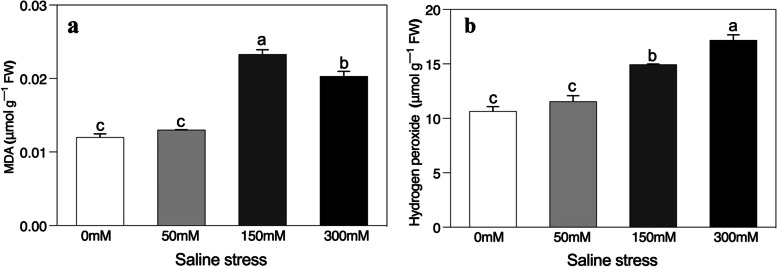
Effects of salinity on the concentration of (**a**) malondialdehyde (MDA), and (**b**) hydrogen peroxide (H_2_O_2_) of *Alhagi sparsifolia* at days 90th of exposure to a different gradient of salinity (0, 50, 150, and 300 mM). Bars represent standard errors of the means (*n* = 3). According to Duncan’s Multiple Range Test, different letters indicate significant differences among the treatments at *P* < 0.05

Saline stress gave rise to significantly higher concentrations of MDA and H_2_O_2_ compared to the controlled conditions (Fig. 4a and b). Interestingly, the maximum concentrations of MDA occurred after the addition of 150 mM and not after 300 mM (Fig. 4a). Moreover, the additions of salt stress significantly increased H_2_O_2_ levels in leaves, relative to the unstressed control, which indicates the persistence of oxidative stress in the studied plants (Fig. 4b).

### Changes in the activities of antioxidant enzymes

**Fig. 5:**
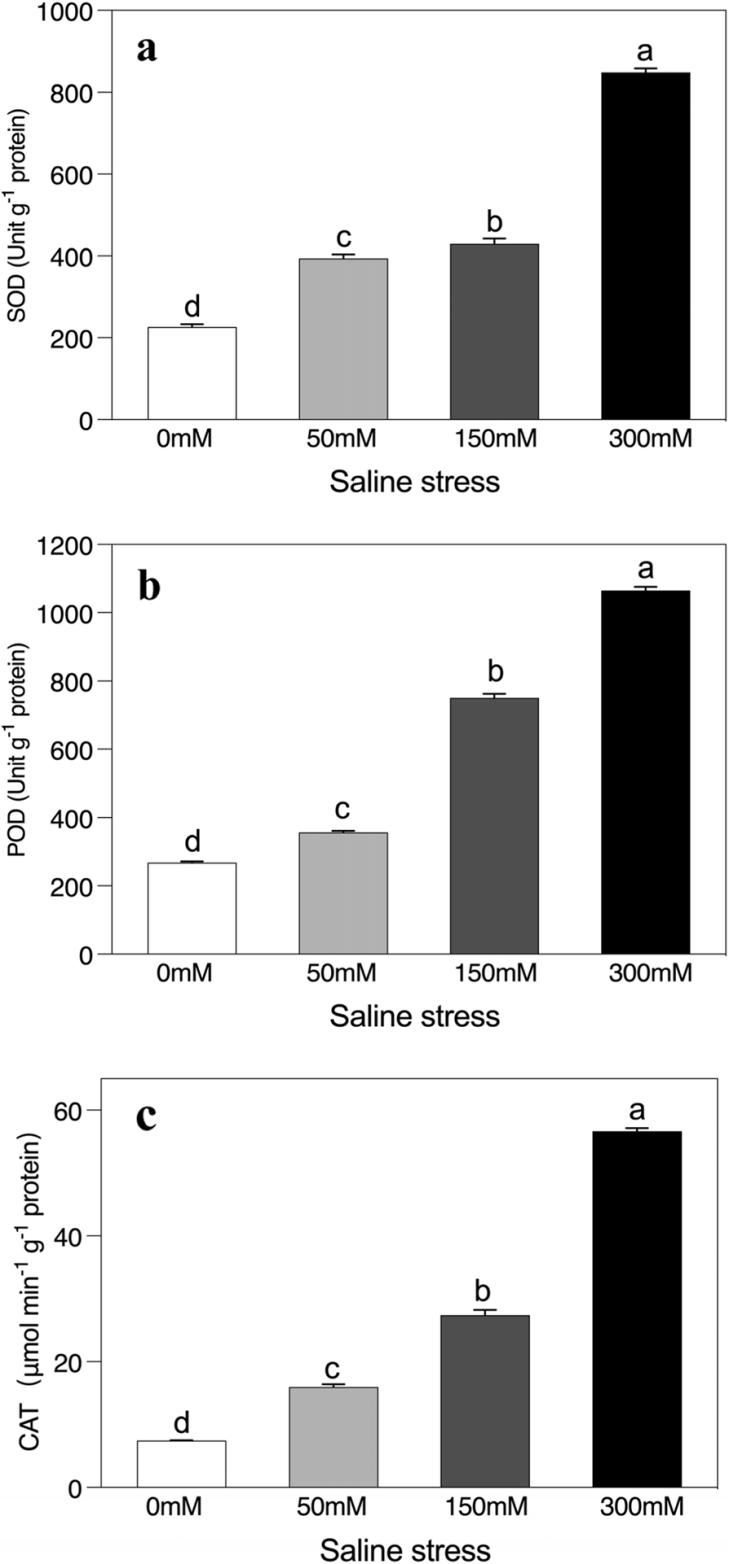
Effects of salinity on the enzymatic activities of (**a**) superoxide dismutase (SOD), (**b**) peroxidase (POD), and (**c**) catalase (CAT) of *Alhagi sparsifolia* at days 90th of exposure to a different gradient of salinity (0, 50, 150, and 300 mM). Bars represent standard errors of the means (*n* = 3). According to Duncan’s Multiple Range Test, different letters indicate significant differences among the treatments at *P* < 0.05

When under stress the scavenging of reactive oxygen species is mainly associated with the production of various antioxidant enzymes that protect plants from severe oxidative damage. Moreover, SOD, POD and catalase activities were gradually enhanced as the saline stress rose, relative to the unstressed control (Fig. 5a-c). For example, SOD, POD and catalase activities increased 3-, 3-, and sevenfold, respectively, when the plants were analysed after the 300 mM saline stress application.

### Salt-stress-dependent changes in carbohydrates, soluble protein and proline concentrations

**Fig. 6:**
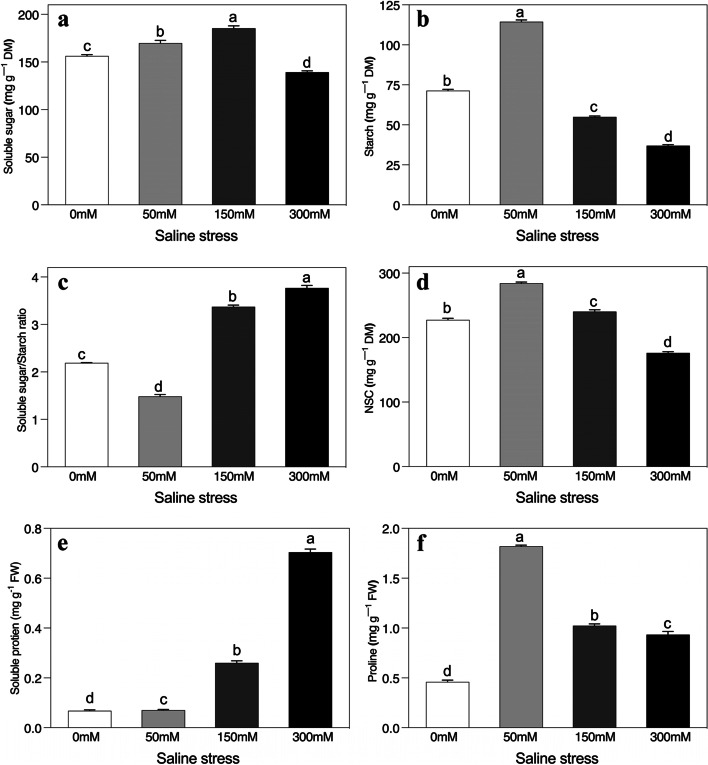
Effects of salinity on the concentration of (**a**) soluble sugar, (**b**) starch, (**c**) soluble sugar/starch ratio, (**d**) nonstructural carbohydrates (NSC), (**e**) soluble protein, and (**f**) proline of *Alhagi sparsifolia* at days 90th of exposure to a different gradient of salinity (0, 50, 150, and 300 mM). Bars represent standard errors of the means (*n* = 3). According to Duncan’s Multiple Range Test, different letters indicate significant differences among the treatments at *P* < 0.05

The concentrations of carbohydrates and proline were also strongly affected by the exposure of *A. sparsifolia* plants to saline stress relative to the unstressed control (Fig. 6a-f). Of the carbohydrates, the soluble sugars, starch and non-structural carbohydrates were all modified in a similar way after the addition of saline stress (Fig. 6a, b, and d). Their values initially increased but then were gradually inhibited as the salt stress intensified relative to the unstressed control. The starch and non-structural carbohydrate concentrations in the 150 mM saline treatment decreased twofold compared to the 300 mM saline treatment (Fig. 6b and d). However, the soluble sugars/starch ratio acted inversely as the saline condition initially inhibited and then gradually enhanced ratio (Fig. 6c), and a twofold increase in saline stress was detected relative to the control. In the case of soluble protein concentrations, salt stress greatly enhanced its production in *A. sparsifolia* and a tenfold difference was recorded between the control and highest saline treatment (Fig. 6e).

Significantly, proline accumulation initially increased vigorously but then declined after the latter two saline additions compared to controlled conditions (Fig. 6f). A fourfold difference existed between the control and 50 mM saline treatment. In contrast, it detected a twofold difference compared to plants with no salt stress in the latter two saline treatments. Finally, compared to the 50 mM saline treatment, a twofold reduction occurred in the 150 mM and 300 mM saline treatments.

### Changes in Na^+^ and K^+^ in leaves and roots

**Fig. 7:**
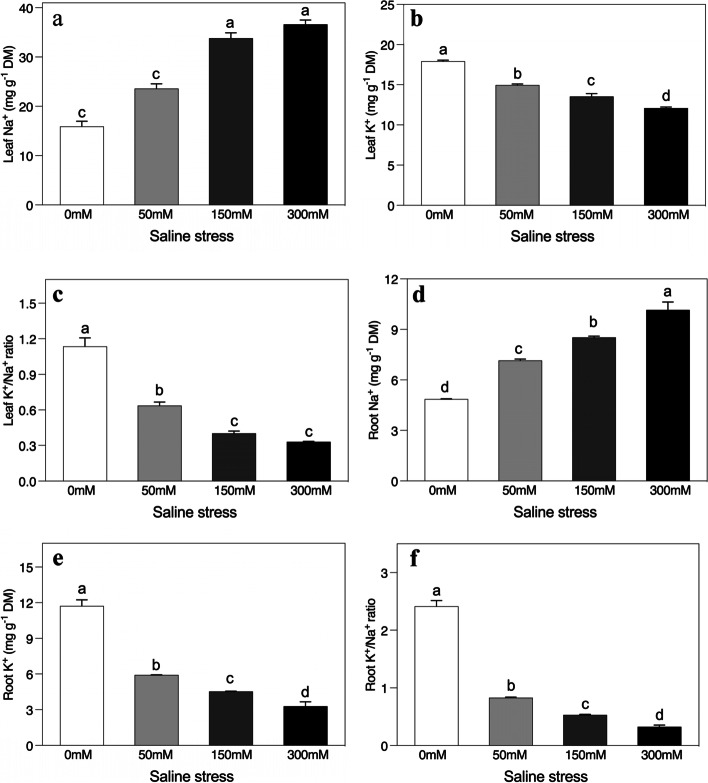
Effects of salinity on the concentration of (**a**) leaf Na^+^, (**b**) leaf K^+^, (**c**) leaf K^+^/Na^+^ (**d**), root Na^+^, (**e**) root K^+^, and (**f**) root K^+^/Na^+^ of *Alhagi sparsifolia* at days 90th of exposure to a different gradient of salinity (0, 50, 150, and 300 mM). Bars represent standard errors of the means (*n* = 3). According to Duncan’s Multiple Range Test, different letters indicate significant differences among the treatments at *P* < 0.05

The imposition of saline stresses significantly unbalanced Na^+^ and K^+^ ions in the leaves and roots of *A. sparsifolia* compared to the controlled conditions (Fig. 7a-f). The increasing concentrations of saline stress significantly enhanced leaf Na + levels, while the K + ion uptake was decreased (Fig. 7a and b). More specifically, when compared to the control, plants showed a twofold increment in leaf Na^+^ ion levels when exposed to the high saline stress (Fig. 7a). Compared to the control plants, the plants treated with 300 mM saline had a threefold inhibition in the K^+^/Na^+^ ratio (Fig. 7c). The root K^+^ ion and Na^+^ ion levels fell or were enhanced, respectively, compared to the controlled conditions. However, differences were much more significant for K^+^ ions (Fig. 7d, e). The same increments in Na^+^ ion levels occurred in roots as in leaves (Fig. 7e). The levels of K^+^ ions were upregulated fourfold in the high saline treatment compared to the control (Fig. 7d) and as a result their ratio had a sevenfold inhibition when plants were exposed to the 300 mM saline addition (Fig. 7f).

### Changes in NO_3_^−^ reduction and NH_4_^+^ assimilation

**Fig. 8:**
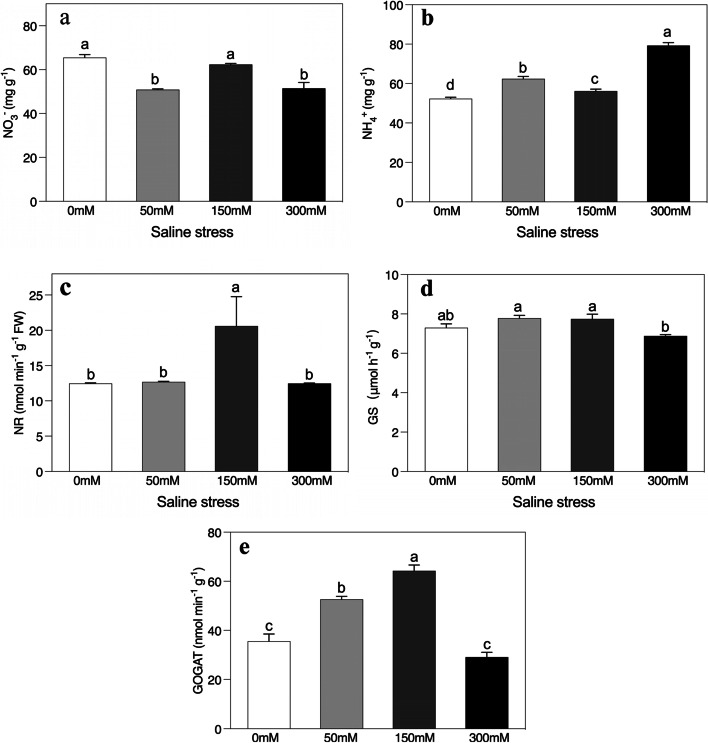
Effects of salinity on the concentration of (**a**) NO_3_^−^ and (**b**) NH_4_^+^ and enzymatic activities of (**c**) nitrate reductase (NR), (**d**) glutamine synthetase (GS), and (**e**) glutamate synthase (GOGAT) of *Alhagi sparsifolia* at days 90th of exposure to a different gradient of salinity (0, 50, 150, and 300 mM). Bars represent standard errors of the means (*n* = 3). According to Duncan’s Multiple Range Test, different letters indicate significant differences among the treatments at *P* < 0.05

The NO^3−^ and NH_4_^+^ concentrations in *A. sparsifolia* plants were notable when exposed to saline stress conditions, relative to the unstressed control (Fig. 8a and b). Specifically, a significant increment was observed in NH_4_^+^ ion levels as the salt stress increased. The high-saline-treated plants had onefold greater NH_4_^+^ ion levels than the untreated plants. The NO_3_^−^ ion levels fell significantly in the 50 mM and 300 mM saline treatments compared to the controlled environment, with the 300 mM saline application exerting the most powerful effect. Interestingly, the addition of 150 mM saline did not provoke any such change. The NR, GS, and GOGAT all reacted remarkably as their activities were initially enhanced but then decreased in the high treatment (Fig. 8c-e). Surprisingly, their (NR, GS, and GOGAT) activities fell significantly after the 300 mM saline application relative to the150mM saline treatment.

### Principal component analysis (PCA)

**Fig. 9:**
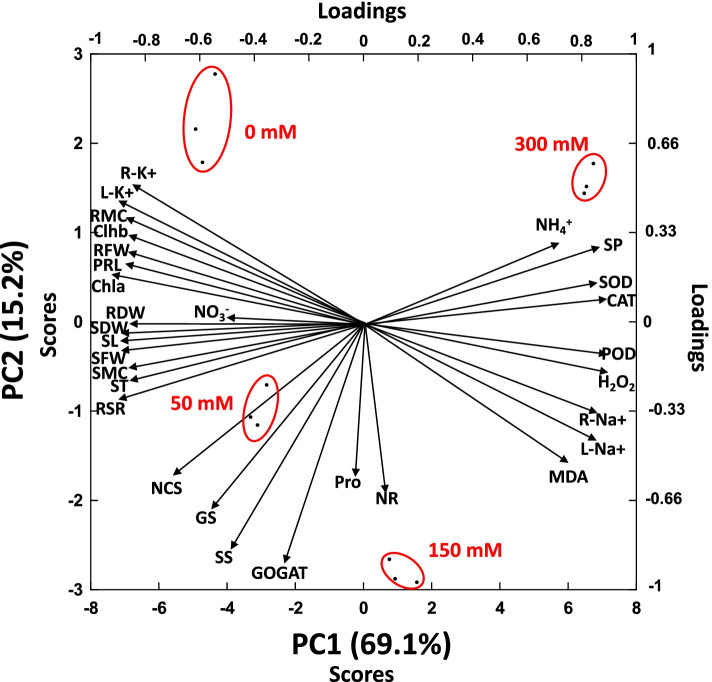
Principal component analysis (PCA) between various growth and physio-biochemical attributes of *Alhagi sparsifolia* at days 90th of exposure to a different gradient of salinity (0, 50, 150, and 300 mM). Acronyms of variables SL = Shoot length, SFW = Shoot fresh weight, SDW = Shoot dry weight, SMC = Shoot moisture content, PRL = Primary root length, RFW = Root fresh weight, RDW = Root dry weight, RMC = Root moisture content, RSR = Root shoot ratio, Chla = Chlorophyll a, Chlb = Chlorophyll b, SP = Soluble protein, MDA = Malondialdehyde, H_2_O_2_ = Hydrogen peroxide, SOD = Superoxide dismutase, POD = Peroxidase activity, CAT = Catalase, NR = Nitrate reductase, GS = glutamine synthetase, GOGAT, P = Proline, SS = Soluble sugar, ST = Starch, NSC = Non-structural carbohydrates, L-K^+^  = Leaf K^+^, L-Na^+^  = Leaf Na^+^, R-K^+^  = Root K^+^, R-Na^+^  = Root Na^+^

The PCA showed along the first PC axis (explaining the 69.1% of total variables variance) that the sets of samples of plants submitted to different levels of salinity had distinct coefficient scores positions in the same order than salinity intensity (Fig. 9). This axis was mainly loaded by plant Na^+^ and K^+^ concentrations, chlorophyll contents, H_2_O_2_ concentrations and enzymes related to ROS elimination. Along PC 2 axis (explaining 15.2% of total variables variance) it was observed that the sample sets of plant growing at intermediate levels of soil salinity were separated by their higher concentrations of proline, enzymes related to N metabolism and sugars contents (Fig. 9).

## Discussion

### Changes in growth and biomass accumulation

The assessment of biomass accumulation in plants exposed to salinity can be used as a cohesive attribute for predicting the responses of plants to salinity stress [43]. According to our study, increasing saline stress decreased the biomass, length, and water content of both shoots and roots of *A. sparsifolia*. A salt-induced reduction in shoot and root growth and biomass attributes has also previously been reported in other plant species [10, 18, 19, 44, 45], supporting our findings. Salt-induced reduction in plant biomass is a crucial survival strategy associated with reduced carbon (C) assimilation [18, 19] as under these conditions, plants allocate more C to energy and maintaining salinity homeostasis (anti-stress mechanisms) than to development and growth [20, 21, 46]. For instance, the biosynthesis and accumulation of stress-relieving biomolecules such as sugar and proline [22–24] is a trade-off with growth, i.e., the resources needed to sustain growth is reduced. Therefore, we can potentially attribute the decrease in growth in *A. sparsifolia* to the allocation of resources by young seedlings to anti-oxidant and osmotic adjustment mechanisms provoked by soil salinity rather than growth. Additionally, the root/shoot ratio also increased (non-significantly) at low salinity levels and belowground biomass dispatch for maintaining a high root-shoot ratio is regarded as one of the key adaptative responses to standing salt stress, which involves directing photosynthetic products to the most limiting organ of the plant [47–49]. The root-shoot ratio then also decreased with salinity levels relative to unstressed control and it has been previously reported that high salt stress reduces the distribution of photoassimilates to the root system [50], leading to a low root-shoot ratio. Therefore, we can suggest that increasing salinity stress reduced the root growth by decreasing the allocation of photoassimilates to roots to establish a possible joint plant mechanism to improve water fluxes from soil to leaves by creating a favorable osmotic pathway.

### Photosynthetic pigments

Variations in chlorophyll concentrations have an impact on plant metabolism [51]. Both chlorophyll a chlorophyll b fell significantly under intermediate and high saline stress similar to other research in salt-stressed plants [52–55]. As a response to salt stress, chlorophyllase activity increases, which leads to a decrease in chlorophyll concentrations as salt-stress levels increases with the extent of the decline in salt stress varying between plant species [56].

The salt-induced response of chlorophyll pigments is recognized as one of several biochemical indicators of salt tolerance in plants [57]. In comparison, increases in the chlorophyll a and chlorophyll a/b ratios in our study were observed at low saline stress which reflects other research that found that a salt-induced increase in chlorophyll concentrations in plants could be attributed to the rise in chloroplast numbers [58, 59]. The results of this research also confirm that salt stress is more detrimental to chlorophyll b than chlorophyll a [60] and this then creates a high chlorophyll a/b ratio since the very first step of chlorophyll b breakdown is its transformation into chlorophyll a [61]. Similar research has suggested that the salt-stress resistance of photosystem II (PSII) and the increased chlorophyll concentrations in plants play an essential role in salt tolerance [58] so it could also be debated that PSII is a crucial element in the salt tolerance of *A. sparsifolia*, although further research on this subject is still needed.

### Ion distribution

Plants under salt stress take up and distribute salt ions via a number of mechanisms [62, 63]. In our study, in agreement with other studies, the accumulation of Na^+^ in root and leaf tissues caused a nutrient imbalance, as shown by the lower K^+^/Na^+^ ratio as salinity stress levels rose that resulted in plant tissues accumulating less dry matter and growing less [64–66]. The concentration of K^+^ may have also fallen antagonistically during the absorption of nutrients due to competition with Na^+^ or to the similarity of its charge. In our study, the saline conditions appears to have impaired high potassium transporters (HKT), which are Na^+^- K^+^ symporters in plants and play an important role in salt tolerance by maintaining K^+^ uptake through K^+^- Na^+^ cotransport [67]. It has been widely speculated that plants under salt stress regulate Na^+^ uptake and transport by maintaining high tissue K^+^/Na^+^ ratios and, therefore, high cytosolic K^+^/Na^+^ ratios, an idea that has become central to salt-tolerance research. K^+^ is crucially important in various physiological processes in plants and in situations of salt stress, high levels of surrounding Na^+^ compete with K^+^ uptake and cause severe K^+^ deficiency and growth impairment [68]. Na^+^ ion-induced outflow of K^+^ from roots and leaves are a significant effect of salt stress on K^+^ regulation [17, 69]. This efflux could potentially be attributed to the excess Na^+^ in the cytoplasm, resulting in lower membrane potential and, consequently, the activation of K^+^ outward rectifier channels, which is thought to be the mechanism by which K^+^ is extruded from cells. Decreasing K^+^ levels in *A. sparsifolia* with increasing saline stress could be the result of a salt-induced inhibition of the ability of cells to maintain a more negative inside potential and so improve intracellular retention of K^+^ (or inhibition of K^+^ efflux) [70]. The ability to retain intracellular K^+^ has also been proposed as crucial for salt stress resistance [71]. In our study, the salt-induced retention of cellular K^+^ decreased with increasing saline stress levels with *A. sparsifolia* retaining cellular K^+^ at low salinity levels but losing this ability as the salt concentrations rise to 150 mM and 300 mM demonstrating that its ability to maintain K^+^ retention is dependent on salt concentrations.

In addition, Na^+^ ions enter roots passively through voltage-independent or faintly voltage-dependent non-selective cation channels so an increase in external Na^+^ will result in a moderate rise in Na^+^ uptake and accumulation, with a consequent reduction in K^+^ uptake [64, 65]. In this study, the concentrations of these ions were higher in leaves than in roots, which has also been reported in other research [59, 72, 73]. Mature *A. sparsifolia* plants in natural deserts have been reported to exhibit a high degree of ion selectivity as a result of arresting excess Na^+^, which keeps salt levels in roots high compared to in leaves [72]. Similarly, other research has also reported that Na^+^ accumulation in tissues of natural mature *A. sparsifolia* plants followed the order root > stem > leaf [74]. Mature *A. sparsifolia* vegetation in natural deserts appears to be most effective at restricting Na^+^ in root tissues whilst in comparison, field-grown *A. sparsifolia* plants form interconnected clones with extensive root systems that probably enable them to dilute and limit excess salt in their roots, which was not possible in our pot experiment with young seedlings. If Na^+^ is transported from roots to shoots when the transpiration stream in the xylem moves [75] it could also be proposed that the increase in leaf Na^+^ and K^+^ ions could be an important survival strategy for juvenile *A. sparsifolia* seedlings where they may use excess ions as osmolytes to decrease the leaf water potential and increase water absorption, and so normalize their photosynthetic capacity.

### Lipid peroxidation, ROS production and antioxidant system

Given its ability to act as indicator of oxidative damage induced by salt stress, lipid peroxidation may be an adequate physiological indicator for evaluating plant stress resulting from both biotic and abiotic factors. This research recorded significantly higher accumulations of MDA and H_2_O_2_ at all levels of saline stress. An increase in leaf H_2_O_2_ could have led to membrane damage in young *A. sparsifolia* due to lipid peroxidation, and to the production of MDA [22, 55, 65]. In addition, ROS not only serve as stress-signalling molecules [76] but also plays an essential role in regulating growth [77] and development [78]. For example, a high H_2_O_2_content should not be viewed simply as a harmful phenomenon to be avoided or alleviated but, instead, also as a necessity for plants needing to respond and adapt effectively and initiate better acclimatization mechanisms [76].

Plants have a highly specialized antioxidant enzymatic defence system responsible for scavenging reactive oxygen species (ROS) at cellular level when under salt stress [34]. In this study, antioxidant enzymes (SOD, POD and CAT) were significantly more abundant in saline-treated *A. sparsifolia* seedlings than in the control. For instance, compared to the control, SOD, POD and CAT increased 3-, 3- and sevenfold, respectively, under 300 mM saline stress indicating that it is a solid antioxidant mechanism for detoxifying ROS [34, 79, 80]. SOD is the first line of defence in the antioxidant defence mechanisms, as it converts the superoxides into H_2_O_2_ and subsequently conversion of H_2_O_2_ into H_2_O and oxygen is performed by CAT. Under stress conditions, low levels of H_2_O_2_ may play a role in signalling [81]. H_2_O_2_-detoxifying enzymes restrict the amount of H_2_O_2_ that cells accumulate rather than eliminate it completely and in addition, POD also scavenges H_2_O_2_ efficiently from the chloroplast [80, 82]. Several studies have reported more SOD, CAT and POD activity in plants subjected to salt stress [45, 65, 80]. However, the relative significance of each antioxidant enzymatic activity (SOD, POD and CAT) needed for scavenging ROS varies from one plant to another in terms of the rates of ROS production and the major sources of ROS, as well as the respective antioxidative activities.

In our study, CAT activity was (sevenfold) greater than POD (threefold) at 300 mM saline stress than in the control, which reflects the greater accumulation of H_2_O_2_ at high salt stress. Generally, the affinity of CAT for H_2_O_2_ is lower than that of POD, although a bulk accumulation of ROS clearly induced CAT activity. With increasing saline stress, *A. sparsifolia* as an adaption mechanism may have increased POD to ensure the cytosolic regulation of H_2_O_2_, whereas the higher CAT activity could be linked to the mass scavenging of H_2_O_2_ [76]. A recent study of *Echinochloa crusgalli* mutants has also demonstrated that POD, APX and GR are involved in fine-tuning ROS production, and that their loss can lead to high levels of ROS, which in turn affects CAT development. CAT is however, unable to reduce H_2_O_2_ to physiological concentrations due to its low affinity so plants suffer from oxidative stress even in the presence of high CAT activity [83]. This study demonstrates that *A. sparsifolia* contains reliable antioxidant mechanisms, both for cytosolic fine-regulation (SOD and POD) and mass-scavenging H_2_O_2_ [60], which could help reduce the detrimental effects of oxidative stress induced by high lipid peroxidation levels. However, a gradual response of these enzymes to gradual increases in saline stress suggests that this plant species has an excellent capacity to adapt to saline stress, including mechanisms especially designed to increase activity when salinity conditions are high.

### Biochemical changes and their role in role in salt mitigation

This study recorded a significant accumulation of proline at all saline stress levels, which may contribute to the protection of the photosynthetic apparatus, the elimination of ROS, and the stabilization of membranes, enzymes, and proteins in salt-stressed young *A. sparsifolia* [84–86]. Additionally, there was a significant increase in soluble sugar at low and intermediate levels, although, compared to the controls, starch content only increased at low levels (Fig. 9). In plants, soluble sugars play several vital roles, including limiting water loss and chlorophyll degradation, regulating cell division, eliminating excess ROS, stabilizing proteins and membrane structure, maintaining osmotic and ionic homeostasis, and controlling transcription of certain genes [22]. Increased salt levels significantly decreased starch accumulation and significantly increasing soluble sugar at 150 mM saline stress (Fig. 9). This might be due to greater activity by the starch-degrading enzymes that mobilize sugars via an intricate network of reactions, as demonstrated in the plant *A. thaliana*, where multiple enzymes work synergistically [87]. The accumulation of starch and soluble sugars and their interrelationships demonstrated in this result that they play a significant role in the salt tolerance of young *A. sparsifolia* at low and intermediate saline stress levels (Fig. 9) [25–27].

High soluble sugar/starch ratio recorded in this study indicate that in the hyperarid saline desert, this could be viewed as an adaptive strategy of young *A. sparsifolia* for synthesizing the enzymes of soluble sugars and avoiding the synthesis of starch enzymes in order to accumulate soluble sugar and adjust the osmotic pressure to maintain water uptake. Proteins also have the potential to act as osmotins and play a part in the development of tolerance to salt stress as identified in other research [11, 88, 89]. In this research, protein concentrations significantly increased in *A. sparsifolia* at all levels of saline stress (low salt < intermediate salt < high salt). Under saline stress, the accumulation of soluble protein in young *A. sparsifolia* may contribute to the osmotic adjustment and to the supply of nitrogen that can be utilized once the salt stress is abated [85, 86].

### Nitrogen assimilation

To ensure salt tolerance, the regulation of nitrogen (N) metabolism is of vital importance, and a complex interplay occurs between salinity and N nutrition [90]. In this study, although, NO_3_^−^ levels fell significantly at 50 mM and 300 mM saline stress, at 150 mM no significant changes relative to the control occurred (Fig. 8b). Lower levels of NO_3_^−^ impede N assimilation and the synthesis of amino acids and proteins, which can result in a fall in plant dry weight [91]. Salt ions also limit NO_3_^−^ reduction by affecting the activity of NR [31, 32, 92]. In contrast to other research this study found that NR activity showed no significant changes at low or high saline stress levels (Fig. 9), and even increased at intermediate saline stress [31, 32]. This could be explained as young plants that have not initiated nodulations have been reported to have less restricted mineral N metabolism due to salinity and they can then germinate and grow in places that have been flooded where soil salt concentrations are lower [93].

It has long been understood that the GS/GOGAT cycle links N and C metabolism in plants by binding inorganic NH_4_^+^ with C skeleton synthesis, a critical step in the metabolism and growth of plants [94]. In this study, GS and GOGAT activities were initially enhanced and then decreased under high saline stress (Fig. 5d, e, and f), indicating that *A. sparsifolia* under low and intermediate saline stress could incorporate NH_4_^+^ effectively to fill its glutamate pool. Even though high salt stress significantly decreased the GS/GOGAT cycle, higher level of soluble proteins and antioxidant enzymes observed in *A. sparsifolia* could be attributed to its N-fixing ability. For instance, the nodulation and biological N_2_ fixation of *A. sparsifolia* has been reported to exhibit high salt tolerance [41, 72]. Under high salinity stress with a retarded GS/GOGAT cycle, *A. sparsifolia* could have the capacity to allocate sufficient energy (sugars) to sustain the bacterial symbionts that ensure a certain level of organic nitrogen for the synthesis of anti-oxidative enzymes.

The assessing of biomass accumulation in plants exposed to salinity can be used as a cohesive attribute for predicting the responses of plants to salinity stress [43]. In this experiment, the imposition of saline stress significantly influenced the biomass and growth attributes of *A. sparsifolia* plants and shoot and root length, shoot and root biomass, and shoot and root water content were significantly inhibited as saline stress increased (Fig. 1). A salt-induced reduction in shoot and root growth and biomass attributes has been previously reported in other plant species [10, 18, 19, 44, 45]. The decrease in growth in *A. sparsifolia* appears to be associated with the allocation of resources by seedlings to anti-oxidant and osmotic adjustment mechanisms provoked by soil salinity rather than to growth.

## Conclusion

The saline stress treatments in our experiment elicited different responses in plant growth and physiological traits. Young *A. sparsifolia* seedlings demonstrated the use of a wide range of salt-tolerant strategies to tolerate low-to-high saline stress, including (i) increased enzymatic anti-oxidant defense mechanism to reduce oxidative stress through the inhibition of ROS and MDA accumulations (ii) enhanced osmolytes synthesis (iii) maintenance of mineral N metabolism (iv) increased chlorophyll a/b ratio and (v), accumulating higher levels of Na + and K + ions in leaves compared to roots that could act as osmolytes to decrease the leaf water potential and increase water absorption for the normalization of metabolism, in hyperarid desert condition. The results strongly suggest a high capacity by *A. sparsifolia* seedlings to manage with low and intermediate-to-high levels of soil salinity; but at very high levels of salinity, the excess of ROS production cannot be fully (or with great difficulty) counteracted and plants can still survive, albeit with reduced growth. Consistent with this observation, were the recorded shifts from more osmolytes adjustment mechanisms to anti-oxidative stress mechanisms, such as the synthesis of anti-oxidative stress enzymes when salinity rose from low-intermediate to high intensity. The results of this research provide a baseline study to improve knowledge of the salt-induced changes in the growth and physiological mechanisms of young *A. sparsifolia* plants in hyperarid saline deserts and potentially assist in future planting of *A. sparsifolia* seeds to improve vegetation restoration in the Taklimakan desert. Further research is required however to understand the underlying physio-biochemical and molecular mechanisms operating under saline stress conditions to increase knowledge of the salt-tolerance capacity of young *A. sparsifolia* plants.

## Materials and methods

A randomized block design (RBD) using pots protected from the rain to control the desired concentration of the applied salt solution was set up as an outdoor experiment in the vicinity of Qira oasis on the southern fringe of the Taklimakan desert (37°00′N, 80°43′E). The annual average temperature in the area is 11.9 °C, with extremes of 41.9 °C and -23.9 °C. The vegetation around the oasis is sparse, with cover of 5–20%, and is composed primarily of shrubs and sub-shrubs such as *A. sparsifolia* and *Tamarix ramosissima.*

We collected *A. sparsifolia* seeds from the desert and planted them in plastic pots (30-cm diameter at the top and 25-cm at the bottom, 25-cm high) filled with 12 kg of soil, and with a hole in the bottom. During the first 50 days, water was supplied to each pot (*n* = 1 seedling) every three days to field capacity (18% w/w) using a weight method. The pots were watered and weighed to replace the evaporated and transpired water. We calculated the soil relative water content (SRWC) using the following formula:

$$SRWC=([(Wsoil-Wpot-DWsoil)/( (\mathrm{WFC}-\mathrm{Wpot}-\mathrm{DWsoil})]*100)$$

In this equation, Wsoil represents the current soil weight (soil + pot + water), Wpot represents the weight of the empty pot, DWsoil the dry soil weight, and WFC the soil weight at field capacity (soil + pot + water). We selected 48 pots with uniform seedlings and divided them into four groups for the application of the stress treatments: three sets of 12 pots for saline stress (NaCl and Na_2_SO_4_, each at a 1:1 molar ratio) and one set of 12 pots for the positive control (0 mM). To avoid osmotic shock, we gradually introduced salt stress by adding 50 mM L^−1^ of saline solution to the stress treatment groups for the first week. Afterwards, saline stress concentrations of 50, 150, and 300 mM were applied. The salt chemicals were mixed in water (500 ml/pot) and applied after every four days. The doses selected were based on real conditions occurring in the field to stimulate saline osmosis stress. Finally, after 90 days of growth, we harvested the leaves of the seedlings and immediately stored them at -80 °C until further analysis (see below).

### Determination of growth parameters

We harvested three randomly selected *A. sparsifolia* seedlings and measured their shoot height, root length, shoot and root fresh weights, shoot and root dry weights, and water content.

### Measurement of photosynthetic pigments

Chlorophyll (0.1–0.3 g fresh leaves) was extracted from the leaves using ethanol (95%, vol/vol) and measured at 665 and 649 nm following the method described by [95]. We calculated the chlorophyll concentrations using the following equations (mg g^−1^ FW):

1 $$\text{Chl a = 13.98 A665 - 6.88 A649}$$

2 $$\text{Chl b = 24.96 A649 - 7.32 A665}$$

3 $$\text{Chl a/b = Chl a / Chl b}$$

### Oxidative stress indicators

Hydrogen peroxide (H_2_O_2_) concentrations were measured using a standardized method [96]. Fresh leaves (0.2 g) were homogenized in 5 ml of trichloroacetic acid (0.1%) in an ice bath, transferred to test tubes, and finally centrifuged at 5000 × g for 10 min (4 °C). The supernatant containing 0.1 mL of titanium reagent (50 µL of 20% titanium tetrachloride) and 0.2 mL of ammonia was centrifuged at 10,000 × g for 10 min. After washing five times with acetone, the precipitate was centrifuged at 10,000 × g for 10 min and then 3 mL of 1 M H_2_SO_4_ was added. The absorbance was read at 410 nm.

Malondialdehyde (MDA) was assessed based on the thiobarbituric acid (TBA) test [97]. Fresh leaves (0.5 g) were homogenized in 1 ml of 5% trichloroacetic acid (TCA) and centrifuged for 10 min at 5000 g (4 °C). In a separate test tube, 4 ml of the supernatant was added to 2 ml of 20% TCA and the mixture was then heated at 100 °C for 15 min and centrifuged at 5000 g (10 min). A spectrophotometer was used to measure absorbance at 450, 532 and 600 nm; the concentrations of MDA were calculated using the following equation:

$$\text{MDA (mol }{\text{g}}^{-1}\text{ FW) = = 6.45 (A532- A600) - 0.56A450.}$$

### Antioxidant enzyme activity

We homogenized the ground leaves in a chilled mortar with 0.1 M phosphate buffer (pH 7.3) with 0.5 mM ethylenediaminetetraacetic acid (EDTA). Afterwards, the homogenate was centrifuged for 10 min at 8000 × g and 4 °C. The activity of SOD was assayed by measuring the reduction rate of nitroblue tetrazolium (NBT) at 560 nm [98]. One unit of SOD activity was defined as the amount of enzyme required for 50% inhibition of NBT reduction at 560 nm.

The POD activity was determined according to standard methods [99, 100], with minor modifications. A reaction mixture was prepared by mixing 2 ml of buffer substrate (8 mM guaiacol and 100 mM Na_3_PO_4_ pH 6.4), 24 mM H_2_O_2_ in 0.5 ml of enzyme extract). At 460 nm, absorbance values were measured twice at 1-min intervals. We calculated enzyme activity by increasing the absorbance of the reaction system by 0.01 up to a maximum of 1U per min, which was then converted into U/g·min − 1. CAT activity was determined by monitoring the disappearance of H_2_O_2_ [101]. Initially, 50 mL of enzyme extract was poured into 1.5 mL of reaction mixture containing 50 mM K-phosphate buffer (pH 7.0) and 15 mM H_2_O_2_. One unit of CAT corresponds to one mole of H_2_O_2_ degradation per minute measured at 240 nm for 1 min. The absorbance was recorded at 240 nm for 1 min. One unit of CAT corresponds to one mole of H_2_O_2_ degradation per min.

### Determination of nitrate assimilation enzymes

Nitrate reductase [102] activity was determined by homogenizing fresh leaves (0.2 g) in 2 mL of 25 mM phosphate buffer saline (PBS, pH 8.7) containing 10 mM cysteine and 1 mM EDTA, which was then centrifuged for 20 min at 30,000 g. The resulting supernatant was tested for NR activity using a diazocoupling method with Griess reagent [103]. The GS activity of frozen leaves was determined by homogenizing them in 2 mL of 50 mM Tris–HCl buffer (pH 7.8; containing 15% glycerol, 0.1% TritonX-100, 1 mM of EDTA and 14 mM of 2-mercaptoethanol) and centrifuging them twice at 4 °C for 10 min. After complexing with acidified ferric chloride, the supernatant was used to determine GS (EC 6.3.1.2) with a 540 nm fluorescence measurement [104]. To determine GOGAT activity, the reaction mixture was composed of 20 mM L-glutamine, 100 mM α-ketoglutaric acid, 10 mM KCl, and 3 mM NADH in 25 mM Tris–HCl (pH 7.6), to which the plant extract was added to initiate the reaction. The absorbance at 340 nm was continuously recorded to monitor the NADH oxidation [104].

### Determination of biochemical parameters

A ball mill was used to grind the dried leaves samples. The colorimetric determination of sugar concentrations was performed by extracting soluble sugar with ethanol according to a standardized method [105]. Starch was measured after perchloric acid extraction (35%; v/v) and analyzed colorimetrically at 620 nm using a slightly modified anthrone method [106]. NSC was obtained by summing the soluble sugar and starch concentrations.

The concentration of Proline (0.2 g fresh levees) was determined using ninhydrin following a standard procedure (105) Toluene was used as a blank for measuring the absorption at 520 nm [107]. Approximately 0.3 g of fresh leaves were used to determine the concentrations of soluble proteins [108], with bovine serum albumin used as the standard. We homogenized 0.2 g of frozen leaves in 5 mL of deionized water to determine NO_3_^−^; for NH_4_^+^ determination, we homogenized 0.2 g of frozen leaves in 2 mL of 10% HCl. A quantitative colorimetric method [109], was used to analyze the supernatants.

### Determination of Na+ and K+

Each dry root and leaf sample (0.05 g) was digested with concentrated HNO_3_ (3 mL). Then the extracts were brought up and adjusted to 15 mL with deionized water. The concentrations of Na^+^ and K^+^ in leaf and root samples were determined using inductively coupled plasma-optical emission spectrometry (ICP-OES), according to a standard method [110].

### Statistical analysis

We repeated all measurements three times and then organized the data with a Microsoft Excel 2019 spreadsheet in terms of the mean values ± SE. The assessment of normality and homogeneity was performed using the Shapiro–Wilk normality test. A one-way analysis of variance was performed with SPSS software (version 13.0; IBM, Armonk, NY, USA). To make pairwise comparisons of the mean values for a given response variable, Duncan’s multiple range test was used with an alpha level of 0.05 for significance. The graphics in the figures were created using Origin Pro 2019 software (Origin Lab Corporation Northampton, MA, USA). To aid interpretation, Pearson correlation analyses (PCA) of the leaf chlorophyll pigment concentrations, N metabolism, osmolytes accumulation, reactive oxygen species production rate, and antioxidant enzymatic activities, as well as root and leaf Na^+^ and K^+^ concentrations, were performed using Origin Pro 2019 software (Origin Lab Corporation Northampton, MA, USA).

To determine the overall relationships of studied plant variables among the samples of plants submitted to distinct intensities of salinity, we used principal component analyses (PCAs) to determine the overall differences of the the studies variables among different salinity levels. We first analysed the data quality by the Kaiser–Meyer–Olkin measure and also eliminated variables with excessive multicollinearity. We conducted one-way ANOVAs with Bonferroni post hoc tests of the scores of the first PC axes to determine differences among the treatments by using Statistica 8.0 (StatSoft, Inc. Tule, Oklahoma, USA).

## Data Availability

All the data generated or analysed during this study are included in this published article.
